# Zearalenone exposure may increase the risk of non-alcoholic fatty liver disease by activating CYP1B1-SCD1

**DOI:** 10.1016/j.crtox.2025.100277

**Published:** 2025-12-13

**Authors:** Haonan Ruan, Jing Zhang, Yunyun Wang, Dan Zhang, Jiaoyang Luo, Meihua Yang

**Affiliations:** a Institute of Medical Genetics and Development, Key Laboratory of Reproductive Genetics (Ministry of Education) and Women’s Hospital, Zhejiang University School of Medicine, Zhejiang 310006, China; b Key Laboratory of Bioactive Substances and Resources Utilization of Chinese Herbal Medicine, Ministry of Education, Institute of Medicinal Plant Development Chinese Academy of Medical Sciences &Peking Union Medical College, Beijing 100193, China

**Keywords:** Zearalenone, Lipid accumulation, Proteomics, Non-alcoholic fatty liver disease, SCD1

## Abstract

Zearalenone (ZEN) is an oestrogen-like mycotoxin that widely contaminates food and feed worldwide. Current research suggests that ZEN causes liver injury by disrupting hepatic lipid metabolism. Furthermore, prolonged disturbances in hepatic lipid metabolism are believed to play a role in the progression of non-alcoholic fatty liver disease (NAFLD). However, the exact association between ZEN exposure and the development of NAFLD is unclear. In this study, we established a rat model of ZEN-induced hepatic lipid accumulation (2.5, 5, 10 mg/kg/d b.w.,14 d, i.g.) to investigate the key targets of ZEN-induced hepatic lipid accumulation and to explore the potential link between ZEN exposure and NAFLD. Histological staining of rat liver showed that ZEN caused significant pathological changes and lipid accumulation; biochemical analysis showed that ZEN significantly and dose-dependently increased serum levels of aspartate aminotransferase (AST) and alanine aminotransferase (ALT) and disrupted the serum lipid profile in rats; proteomic analysis showed that ZEN significantly altered protein expression levels in rat liver, especially SOAT1, SOAT2, CYP1B1 and SCD1 (p < 0.05). Through bioinformatics analyses, we combined ZEN exposure with a database of NAFLD clinical samples to reveal that ZEN exposure may increase the risk of NAFLD through activation of the CYP1B1-SCD1 pathway, and further animal experiments confirmed this. This study identifies the key target of ZEN-induced lipid accumulation and provides a basis for further research on the intrinsic link between ZEN exposure and NAFLD.

## Highlights

• Exposure to the ZEN induces dose-dependent hepatotoxicity and promotes hepatic lipid accumulation in rats.
• Proteomic analysis revealed that ZEN exposure upregulates endoplasmic reticulum stress-related enzymes in rat liver.
• Toxicological and clinical data demonstrates that ZEN exposure may increase the risk of NAFLD by activating CYP1B1-SCD1.

## Graphical abstract

**Figure:**
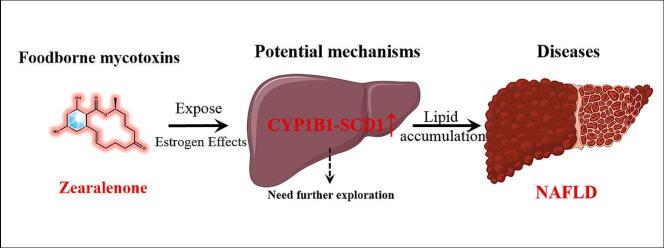

## 1 Introduction

ZEN is a mycotoxin that is produced by various species of *Fusarium* fungi, and is commonly found as a contaminant in grains, such as corn, wheat, barley and oats (Rai et al., 2020). A report published by Nature Food in 2022 suggests that ZEN poses a significant public health risk as one of the most important *Fusarium* toxins contaminating European wheat (Johns et al., 2022). ZEN has drawn significant attention due to its potential harmful effects on human and animal health. Current studies have shown that ZEN causes varying degrees of reproductive toxicity (Wang et al., 2022a), hepatotoxicity (Wu et al., 2023) and immunotoxicity (Ruan et al., 2023a). Due to the high contamination rate and toxicity of ZEN, almost all countries or organisations including the EU and FDA have set strict limits for ZEN in food and feed (E Commission, 2006).

Current research indicates that ZEN could induce liver injury through multiple mechanisms, including the activation of the cytochrome P450 (CYP450) enzyme system, induction of DNA damage, and triggering of inflammatory responses (Ruan et al., 2022). Recent studies showed that ZEN exposure could lead to metabolic disruption and changes in circulating adaptines concentrations in pigs (Nagl et al., 2021). The metabolomics analysis of mouse liver showed that ZEN exposure resulted in significant changes in the concentration of 91 metabolites (including lipids and lipid molecules) (Li et al., 2021). ZEN exposure could also lead to lipid accumulation in zebrafish liver, and further transcriptomic analysis showed significant enrichment of lipid peroxidation pathways (Zhang et al., 2022). These studies indicated that exposure to ZEN can cause liver damage by disrupting liver lipid metabolism.

Abnormal hepatic lipid metabolism is a key contributor to various liver diseases, particularly NAFLD. As the most prevalent chronic liver condition globally, NAFLD etiology remains incompletely understood, though it is thought to involve epigenetic, dietary, and environmental factors (Loomba et al., 2021). A study suggested that lipid metabolism disorders caused by exposure to mycotoxin might induce NAFLD (Bechynska et al., 2021). Ochratoxin A could induce liver steatosis through the PPARγ-CD36 axis, indicating that the impact of Ochratoxin A on liver lipid metabolism might lead to the development of NAFLD (Zheng et al., 2021). Deoxynivalenol, which belongs to the same category of *Fusarium* toxin as ZEN, could cause imbalances in liver hormones and metabolism, and lead to liver steatosis by altering the expression of genes related to lipid oxidation, adipogenesis and lipolysis, thereby inducing NAFLD (Barbouche et al., 2020). Experimental and epidemiological data indicated that environmental exposure to ZEN played a significant role in the development and progression of NAFLD (De et al., 2025).The latest research showed that ZEN exacerbates lipid metabolism disorders by promoting liver lipid droplet formation in mice (Han et al., 2025). Meanwhile, ZEN exposure exacerbated accumulation of hepatic lipid droplets of mice under a high-fat diet state (Wang et al., 2022b). Therefore, we hypothesized that a potential association may exist between ZEN-induced hepatic lipid accumulation and NAFLD.

Through proteomic and bioinformatic analyses, we discovered that CYP1B1-SCD1 may be a potential key target linking ZEN exposure to NAFLD risk. CYP1B1 was a key enzyme in the metabolism of estrogen (White et al., 2012). Current research has found that CYP1B1-SCD1 axis plays a critical role in metabolic diseases such as obesity and NAFLD (Li et al., 2017). Microarray gene expression which was analyzed in livers of normal and congenic CYP1B1-ko mice fed either low or high fat diets, indicated that CYP1B1 could affect liver fat homeostasis in mice by regulating its downstream target SCD1 (Bushkofsky et al., 2016). Current study demonstrated that CYP1B1 played an important role in fatty acid metabolism and that CYP1B1-SCD1 inhibition significantly attenuated high-fat diet-induced NAFLD, improved glucose tolerance and prevented hepatic steatosis (Larsen et al., 2015, Liu et al., 2015). Lipidomics studies have demonstrated that CYP1B1 influenced liver lipid homeostasis by regulating SCD1 and the lysophosphatidylcholine pathway (Li et al., 2014). A clinical study suggested that downregulation of SCD1 can effectively alleviate the occurrence and development of NAFLD (Ratziu et al., 2021). Based on this, we hypothesize that ZEN exposure may increase the risk of NAFLD by activating the CYP1B1-SCD1 axis.

Current research has shown that exposure to ZEN can lead to liver damage and abnormal hepatic lipid accumulation, but the potential link between ZEN and NAFLD is still unclear. In order to further investigate the toxic effects of ZEN on the liver and to identify its key targets, this study established an animal model of ZEN-induced hepatic lipid accumulation in rats. Firstly, we evaluated the hepatotoxic effects of ZEN and its impact on hepatic lipid accumulation in rats through multiple established methods, including the analysis of liver coefficients, hematoxylin and eosin (HE) staining of liver tissues, measurement of serum biochemical parameters, and oil red O staining to assess lipid deposition; Secondly, we performed proteomics analysis on the livers of each group to search for the key targets of ZEN-induced hepatic lipid accumulation; Then, we performed bioinformatics analysis of the differential genes of ZEN exposure and the differential genes of NAFLD clinical samples to initially explore the potential link between ZEN exposure and NAFLD based on the latest database. Finally, western blotting and immunofluorescence (IF) assays were performed to confirm the activation of signaling pathways induced by exposure to ZEN. This study provides a basis for exploring the molecular mechanism of ZEN-induced lipid accumulation in rat liver and provides direction for the prevention and treatment of NAFLD from the perspective of mycotoxin exposure.

## 2 Materials and methods

### 2.1 Chemicals and reagents

ZEN was obtained from Pribolab (Qingdao, China). Carboxymethylcellulose sodium, ethyl carbamate and paraformaldehyde was obtained from Yuanye (Shanghai, China). Ethanol, acetic acid and hydrochloric acid were obtained from Sigma-Aldrich (Shanghai, China). Hematoxylin eosin staining kit, AST Assay Kit, ALT Assay Kit and BCA Protein Assay Kit, Triglyceride (TG) Content Assay Kit, Total Cholesterol (TC) Content Assay Kit, Low-Density Lipoprotein Cholesterol (LDL-C) Content Assay Kit and High-Density Lipoprotein Cholesterol (HDL-C) Content Assay Kit were obtained from Solarbio (Beijing, China). Anti-CYP1B1 and anti-SCD1 were obtained from Abcam (Shanghai, China).

### 2.2 Animal study

All experimental procedures involving animals were conducted in accordance with the Guide for the Care and Use of Laboratory Animals as promulgated by ARRIVE. The study received ethical approval from the Laboratory Animal Center of the Institute of Medicinal Plant Development, Chinese Academy of Medical Sciences and Peking Union Medical College (Approval No. SLXD-20210422034). Rats offer an optimal balance of physiological relevance, research feasibility, data comparability, and cost-effectiveness, making them the preferred and classic animal model for studying ZEN-induced hepatic lipid accumulation and other toxicological effects (Oser, 1981, Xu et al., 2025, Yuan et al., 2024).

Twenty male Sprague-Dawley (SD) rats, aged 8 weeks and weighing 200 ± 20 g, were supplied by Beijing Vital River Laboratory Animal Technology Co., Ltd. The animals were housed under controlled environmental conditions, including a 12-hour light/dark cycle, constant temperature of 23 ± 3 °C, and relative humidity of 50 ± 10 %. Standard nutrition was provided through UV-sterilized fodder (NCD, Beijing Hfk Bioscience Co., Ltd., Beijing, China) and water available ad libitum. Prior to formal experimentation, all rats underwent a one-week acclimatization period. In strict adherence to the guideline-specified exclusion criteria, no animals, experimental units, or data points were removed from the study.

The rats were randomly assigned to four groups: control group (n = 10), ZEN-L group (n = 10), ZEN-M group (n = 10) and ZEN-H group (n = 10). Rats in the ZEN-L, ZEN-M and ZEN-H group were administered ZEN via oral gavage at a dose of 2.5, 5,10 mg/kg body weight per day, dissolved in 0.3 % sodium carboxymethyl cellulose (CMC-Na) respectively; The control group received an equal volume of the vehicle (0.3 % CMC-Na) following the same schedule. The treatment continued for 14 days (Ruan et al., 2023b). Throughout the experiment, all animals had free access to food and water, with daily monitoring of body weight and food consumption. After the final administration, rats were fasted for 16 h and subsequently anesthetized with an intraperitoneal injection of ethyl carbamate (20 %, 1 g/kg). Blood samples were collected and allowed to clot at room temperature for 30 min, followed by centrifugation at 2000 × g for 10 min at 4 °C to isolate serum. Euthanasia of rats after blood collection. Livers were immediately excised and placed on ice, weighed, and rinsed with ice-cold saline. Each liver was divided into two portions: one was fixed in 4 % paraformaldehyde for histological analysis, and the other was snap-frozen in liquid nitrogen and stored at −80 °C for further biochemical assays.

### 2.3 HE staining

HE staining was performed as described previously (Li et al., 2019). Fresh tissue samples were fixed in 4 % paraformaldehyde for 48 h, followed by trimming and placement into embedding cassettes. The tissues were subsequently dehydrated through a graded series of ethanol (70 %, 80 %, 95 %, and 100 %), spending 30 min in each concentration. This was followed by clearing in two changes of xylene, 20 min each, and infiltration with paraffin using two baths of molten wax, 12 min per bath. Embedding was performed using a paraffin embedding station. Sections were cut at a thickness of approximately 4  μm using a microtome. The resulting paraffin sections were then subjected to HE staining. The staining procedure involved deparaffinization in three changes of xylene (8 min each), rehydration through a graded alcohol series (two changes of 100 % alcohol, 90 %, 80 %, and 60 % alcohol, 8 min each), staining with hematoxylin for 4 min, rinsing in running water, differentiation in hydrochloric acid-alcohol for 2–3 s, and bluing in 90 % alcohol for 3–5 s. This was followed by counterstaining in eosin, dehydration through 95 % alcohol (5 min) and three changes of 100 % alcohol (5 min each), and final clearing in xylene. The stained sections were mounted with neutral resin and examined under a light microscope equipped with an imaging system for evaluation of histopathological changes.

### 2.4 Oil red O staining

Oil red O staining was conducted following an established protocol (Benegiamo et al., 2018). In brief, rats were euthanized and the liver was perfused with ice-cold 1 × PBS to remove residual blood. Tissue sections from the major liver lobes were embedded in Optimal Cutting Temperature (O.C.T.) compound (Tissue-Plus, Scigen, 4583) and stored at − 80 °C. For cryosectioning, the embedded tissues were equilibrated to − 20 °C in a cryostat chamber, and 14 μm-thick sections were prepared. The unfixed tissue sections were stained with Oil Red O, and images were captured using a Zeiss VivaTome microscope at 20 × magnification. Ten images were acquired per animal, and the hepatic lipid content was quantified using ImageJ software.

### 2.5 Determination of biochemical indices in rat serum

Serum levels of AST, ALT, TC, TG, LDL-C and HDL-C in rats were measured using commercial assay kits according to the manufacturer’s instructions, following the methodology as previously described (Kang et al., 2021). Briefly, serum samples were directly applied to the assay without pretreatment. Absorbance values were recorded using a microplate reader, adhering strictly to the procedural guidelines provided with the kits. A standard curve was constructed using the values obtained from the standard solutions provided in the kit. The concentration of each standard solution was plotted on the x-axis, while the corresponding ΔA (absorbance difference between the standard and blank tubes) was plotted on the y-axis. A linear regression equation (y = kx + b) was derived from the standard curve. The AST, ALT, TC, TG, LDL-C and HDL-C concentrations in the serum samples were then determined by substituting the ΔA values of each sample (A assay tube – A control tube) into the regression equation to solve for x.

### 2.6 Proteomic analysis of rat liver

Proteomic and bioinformatics analyses of rat livers were performed according to standard operating procedures as previously described (Wang et al., 2022c). Liver tissue samples from rats were subjected to pretreatment, and the total protein concentration was quantified using a BCA assay kit. Following pretreatment, the samples underwent enzymatic digestion and were subsequently desalted with a C_18_ solid-phase extraction column. The resulting peptides were separated by an EASY nLC 1200 ultra-high performance liquid chromatography system (ThermoFisher Scientific, Waltham, MA, USA) and ionized via nanoelectrospray for analysis on a Q Exactive™ HF-X mass spectrometer (Thermo Fisher Scientific). The acquired MS/MS spectra were processed and identified using the MaxQuant software (version 1.6.15.0).

### 2.7 Gene expression data set

Differentially expressed genes (DEGs) associated with ZEN exposure were retrieved from two major databases: GeneCards (https://www.genecards.org/) (Safran et al., 2010) and the Comparative Toxicogenomics Database (CTD; https://ctdbase.org/) (Davis et al., 2023). Additionally, genes corresponding to differentially expressed proteins (DEPs) identified in our proteomic analysis were incorporated into the ZEN-related gene set. For NAFLD-associated genes, raw transcriptomic datasets were downloaded from the Gene Expression Omnibus (GEO) under accession number GSE89632 (Arendt et al., 2015). These data were derived from liver tissue samples of both NAFLD patients and healthy controls.

### 2.8 Identification of DEGs

The acquired data were processed in RStudio (version 3.6.2) using multiple R packages. In accordance with the methodology of the original study, each dataset underwent quality control to reduce false positives. Normalization of the expression matrix was performed with the normalizeBetweenArrays function from the limma package (version 3.42.2) (Ji et al., 2021). Differential expression analysis was subsequently carried out using the limma package to compare both ZEN-exposed samples against control-treated samples, and NAFLD samples against matched controls. Genes meeting the criteria of p-value < 0.05 and |log_2_ fold change| ≥ 1.2, as established in the original study, were defined as differentially expressed genes (DEGs).

### 2.9 Western blot of rat liver

Western blot of rat livers were performed according to standard operating procedures as previously described (Baksh et al., 2020). Whole tissue samples were lysed in RIPA buffer supplemented with protease and phosphatase inhibitors. Thirty micrograms of total protein per sample were separated by electrophoresis using a pre-cast stacking gel and subsequently transferred onto PVDF membranes via electroblotting. The membranes were probed with primary antibodies, followed by washing and incubation with horseradish peroxidase (HRP)-conjugated secondary antibodies—either goat anti-rabbit IgG or goat anti-mouse IgG-at a dilution of 1:10,000. Antibody dilutions used were as follows: anti-CYP1B1 (1:1000) and anti-SCD1 (1:1000). Signal detection was performed using a chemiluminescent substrate, and automated image acquisition was carried out following exposure to enhanced chemiluminescence solution.

### 2.10 IF of rat liver

IF of rat livers were performed according to standard operating procedures as previously described (Maller et al., 2021). Fresh tissue samples from the livers were fixed in 4 % paraformaldehyde for 48 h, followed by paraffin embedding and sectioning. The sections were dewaxed and rehydrated, after which endogenous peroxidase activity was quenched. Antigen retrieval was performed using citrate buffer at either pH 7 or pH 9 (DAKO). After blocking, the sections were incubated overnight at 4 °C with primary antibodies against CYP1B1 and SCD1 (diluted 1:50). This was followed by a 1-hour incubation at room temperature with HRP-conjugated goat anti-rabbit secondary antibody (1:2000). Finally, the slides were counterstained with DAPI, mounted, and visualized under an inverted fluorescence microscope.

### 2.11 Statistical analyses

Statistical analysis was conducted using GraphPad Prism software (version 8.0.2). Data were collected as repeated measurements from independent biological replicates and are presented as mean ± standard deviation. Differences between groups were evaluated using the Student’s *t*-test, with a p-value of less than 0.05 considered statistically significant and a p-value below 0.01 deemed highly statistically significant.

## 3 Results

### 3.1 ZEN exposure leads to dose-dependent lipid accumulation in rat liver

**Fig. 1:**
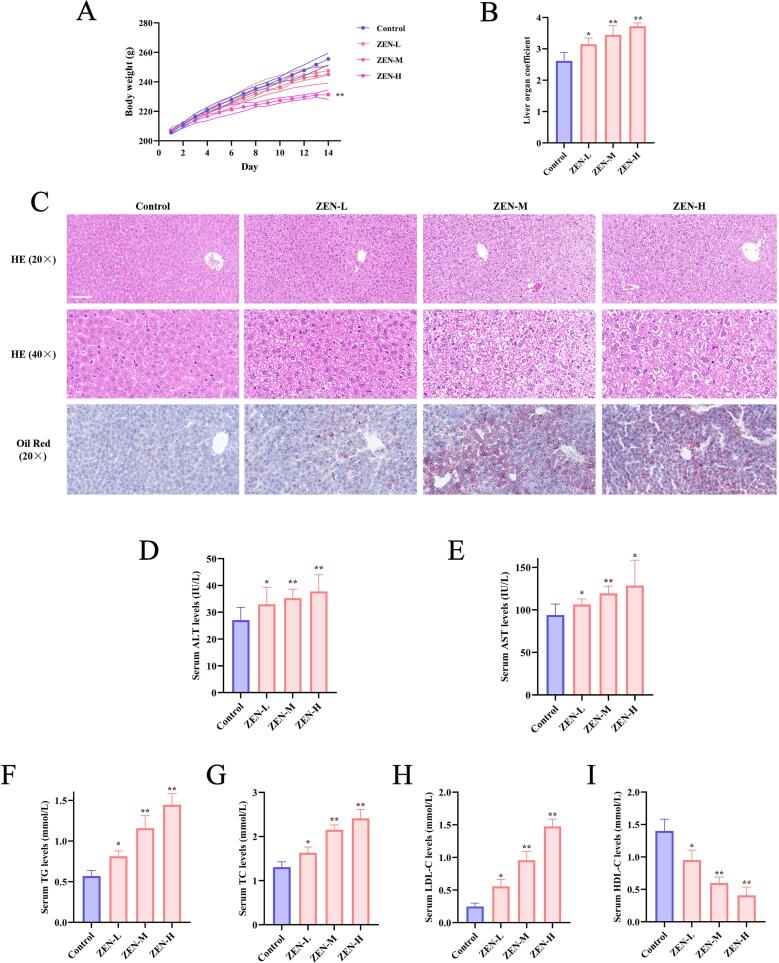
The pathological phenotypes in rats from control, ZEN-L, ZEN-M, and ZEN-H groups. (A) Body weight of rats in control, ZEN-L, ZEN-M, and ZEN-H groups; (B) Liver coefficient of rats in control, ZEN-L, ZEN-M, and ZEN-H groups. (C) Representative images of HE and oil red O staining of rat liver in control, ZEN-L, ZEN-M, and ZEN-H groups, scale: 200 μm; (D) Serum ALT in control, ZEN-L, ZEN-M, and ZEN-H groups; (E) Serum AST in control, ZEN-L, ZEN-M, and ZEN-H groups.(F) Serum TG in control, ZEN-L, ZEN-M, and ZEN-H groups. (G) Serum TC in control, ZEN-L, ZEN-M, and ZEN-H groups. (H) Serum LDL-C in control, ZEN-L, ZEN-M, and ZEN-H groups. (I) Serum HDL-C in control, ZEN-L, ZEN-M, and ZEN-H groups; Data are expressed as mean ± SD. Statistical significance was determined by two-sided unpaired Student’s *t*-test. n = 10. *p < 0.05, **p < 0.01 as compared to the control group.

In order to study the effect of ZEN on rat liver, we compared and analysed the body weight, liver coefficients, HE staining, Oil red O staining and serum indices of rat in ZEN and control groups. Compared to the control group, there was no significant difference in the body weight of rats in the ZEN-L and ZEN-M groups. However, the body weight of rats in the ZEN-H group was significantly reduced, indicating a certain degree of toxicity (Fig. 1A) (p < 0.01). ZEN exposure significantly increased the liver coefficients in rats in a dose-dependent manner compared to control group (Fig. 1B) (p < 0.01). In HE staining examination of histological sections of liver pathology, the ZEN-L, ZEN-M and ZEN-H group showed hepatomegaly, cytoplasmic laxity, lymphocytic infiltration, granular degeneration and severe vesicular degeneration of hepatocytes as compared to the control group. Oil red O staining results indicated that, compared with the control group, ZEN exposure promoted lipid accumulation in rat livers in a dose-dependent manner (Fig. 1C). Consistent with the trend of altered liver coefficients, the serum levels of ALT (Fig. 1D) (p < 0.01) and AST (Fig. 1E) (p < 0.01) were significantly higher in the ZEN-L, ZEN-M and ZEN-H group of rats than in the control group. The lipid profile results showed that ZEN exposure significantly elevated serum levels of TC (Fig. 1F) (p < 0.01), TG (Fig. 1G) (p < 0.01), and LDL-C (Fig. 1H) (p < 0.01), while reducing the level of HDL-C (Fig. 1I) (p < 0.01) in a dose-dependent manner. In summary, our experimental results demonstrate that ZEN exposure significantly promotes hepatic lipid accumulation and induces liver injury in a dose-dependent manner in rats.

### 3.2 ZEN exposure alters the proteomics of the rat liver

**Fig. 2:**
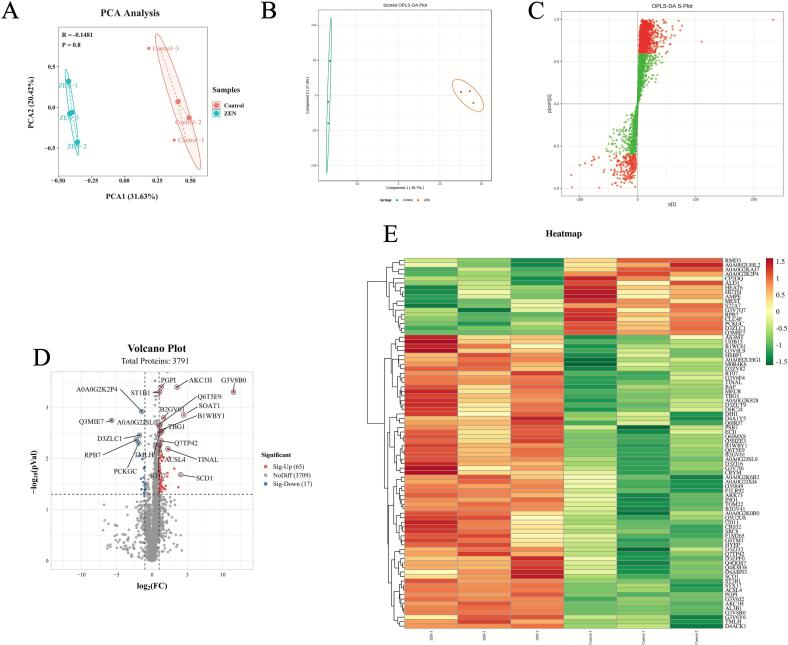
The PCA analysis, OPLS − DA analysis, volcano plot and clustering heatmap of proteomic. (A) The PCA analysis of proteomic in control and ZEN group; (B) The OPLS − DA analysis of proteomic in control and ZEN group; (C) S − plot of OPLS − DA analysis; (D) The volcano plot of proteomic; (E) The clustering heatmap of proteomic. Data are expressed as mean ± SD. Statistical significance was determined by two-sided unpaired Student’s *t*-test. n = 3.

To further explore the key targets of ZEN exposure-induced lipid accumulation, we conducted comparative analysis and profiling of the liver proteome in control and ZEN-M group rats. PCA (Fig. 2A) and OPLS-DA (Fig. 2B,C) analysis was employed to distinguish the proteomes of the two groups, yielding favorable model parameters (R^2^X = 0.631, R^2^Y = 0.997, Q^2^ = 0.798). By combining log_2_ Fold Change (FC) and p-value criteria (|log_2_FC| > 1.0, p-value < 0.05), we identified 82 differentially expressed proteins (DEPs) in the ZEN group, consisting of 65 upregulated and 17 downregulated proteins (Fig. 2D). We further performed cluster heatmap analysis using these DEPs (Table S1) (Fig. 2E). The proteomic analysis comparing ZEN and control groups revealed significant alterations in protein expression levels in the rat liver induced by ZEN exposure.

### 3.3 ZEN increases the expression level of SOAT family protein in rat liver

**Fig. 3:**
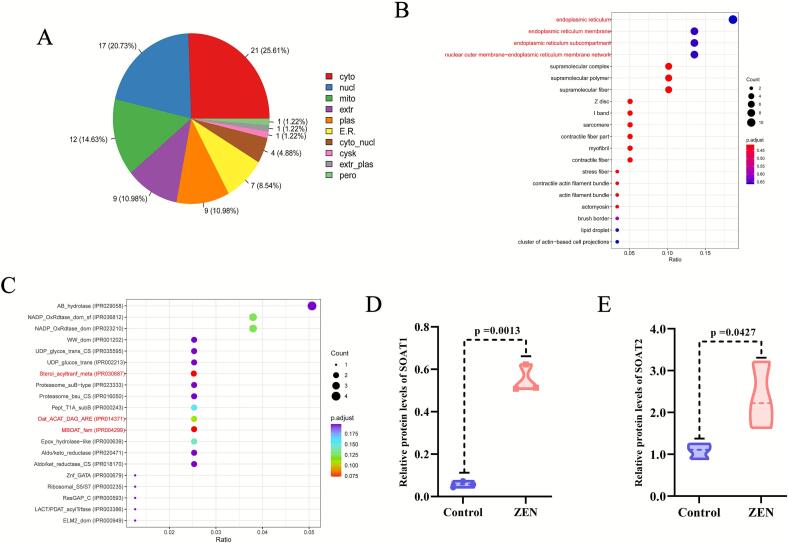
Subcellular localization, GO analysis of cell component enrichment analysis and domain enrichment analysis. (A) Subcellular localization of proteomic; (B) GO analysis of cell component enrichment analysis of proteomic; (C) Domain enrichment analysis; (D) Violin plots of SOAT1 between control and ZEN group; (E) Violin plots of SOAT2 between control and ZEN group. Data are expressed as mean ± SD. Statistical significance was determined by two-sided unpaired Student’s *t*-test. n = 3.

After determining the significant changes in rat liver proteomics caused by ZEN exposure, in order to further explore its key targets, we conducted subcellular localization analysis, domain enrichment analysis and GO enrichment analysis on DEPs from ZEN and control groups. The subcellular localization analysis revealed that 25.61 % of DEPs were located in the cytoplasm, it is noteworthy that DEPs located in the endoplasmic reticulum account for 8.54 % (Fig. 3A). Consistent with subcellular localization, GO analysis of cell component enrichment showed that DEPs were highly enriched in cell components such as endoplasmic reticulum, endoplasmic reticulum membrane, endoplasmic reticulum subcompartment and nuclear outer membrane-endoplasmic reticulum membrane network (Fig. 3B). The structural domain enrichment analysis revealed that the DEPs were primarily associated with Sterol-acyltranf-meta, Oat-ACAT-DAG-ARE and MBOAT-fam structural domains (Table 1) (Fig. 3C). Our experimental results showed that compared with the control group, ZEN exposure significantly increased the protein expression levels of SOAT1 (Fig. 3D) (p < 0.01) and SOAT2 (Fig. 3E) (p < 0.05) in the rat liver, with ZEN exposure being more sensitive to the impact of SOAT1 protein expression.

**Table 1:** Key DEPs of ZEN vs. control. in Rat liver.

**Proteins**	**Control-1**	**Control-2**	**Control-3**	**ZEN-1**	**ZEN-2**	**ZEN-3**	Log_2_FC*	p-value*
G3V7I6_SOAT2	0.8912	1.1089	1.2532	1.6349	2.2209	3.2128	1.1196	0.0427
CP1B1_CYP1B1	0.0922	0.1115	0.0403	0.3314	0.3103	0.2519	1.873	0.0402
G3V6J2_SOAT1	0.0728	0.0573	0.0434	0.5132	0.5085	0.6227	3.1386	0.0158
SCD1	0.0589	0.0835	0.1366	1.0285	3.0161	0.53	4.0353	0.0208

*|Log_2_FC| > 1.0, p-value < 0.05 were considered statistically significant.

### 3.4 ZEN exposure affects the expression levels of CYP1B1 and SCD1 proteins in rat liver

**Fig. 4:**
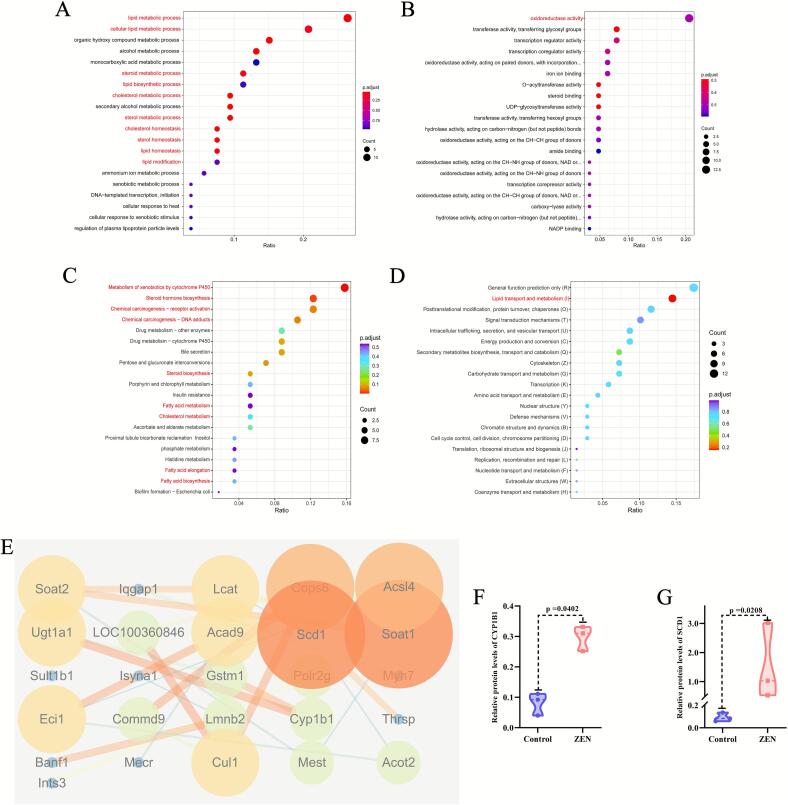
GO enrichment analysis, KEGG enrichment analysis, KOG enrichment analysis and PPI analysis of proteomic. (A) The biological process enrichment analysis of GO analysis; (B) The molecular function enrichment analysis of GO analysis; (C) The KEGG enrichment analysis of proteomic; (D) The KOG enrichment analysis of proteomic; (E) PPI analysis of proteomic; (F) Violin plots of CYO1B1 between control and ZEN group; (F) Violin plots of SCD1 between control and ZEN group. Data are expressed as mean ± SD. Statistical significance was determined by two-sided unpaired Student’s *t*-test. n = 3.

To further explore the specific mechanism of ZEN exposure leading to liver toxicity in rats, we performed Cluster of Orthologous Groups (KOG) and Kyoto Encyclopedia of Genes and Genomes (KEGG) enrichment analyses, as well as protein–protein interaction (PPI) analysis, on the DEPs between the ZEN and control groups. In the biological process enrichment analysis of Gene Ontology (GO) analysis, we observed that the DEPs were primarily involved in lipid metabolism processes, including cellular lipid metabolic process, steroid metabolic process, lipid biosynthetic process, cholesterol metabolic process, sterol metabolic process, cholesterol homeostasis, sterol homeostasis, lipid homeostasis and lipid modification (Fig. 4A). Notably, the molecular function enrichment analysis of GO analysis encompassed oxidoreductase activity, transferase activity, O-acyltransferase activity and steroid binding (Fig. 4B). KEGG enrichment analysis of the DEPs demonstrated that ZEN exposure significantly affected metabolism of xenobiotics by cytochrome P450, steroid hormone biosynthesis, steroid biosynthesis, fatty acid metabolism and cholesterol metabolism (Fig. 4C). KOG enrichment analysis suggested that the liver toxicity induced by ZEN exposure was mainly related to lipid transport and metabolism (Fig. 4D).

Combining the above analysis and PPI analysis (Fig. 4E), it is suggested that the key DEPs enriched in lipid metabolism related pathways were CYP1B1, SCD1 (TOP2 DEPs), SOAT1, and SOAT2 proteins (Table 1). In our study, ZEN exposure significantly upregulated the protein expression levels of CYP1B1 (Fig. 4F) (p < 0.05) and SCD1 (Fig. 4G) (p < 0.05) in rat liver compared to the control group, and PPI analysis (Fig. 4E) suggested that SCD1 is one of the proteins with the highest degree and correlation.

### 3.5 ZEN exposure may increase the risk of NAFLD by activating CYP1B1-SCD1

**Fig. 5:**
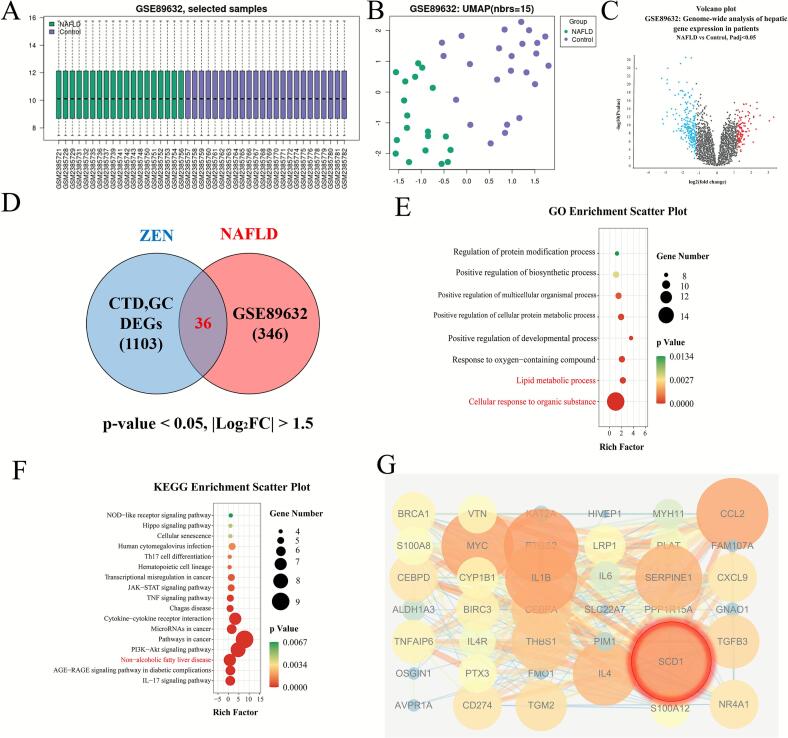
Bioinformatics analysis of ZEN exposure and NAFLD. (A) Histogram of the distribution of NAFLD patient samples and healthy population samples; (B) Scatter plot of sample distribution; (C) The volcano plot of GSE89632; (D) The Venn diagram of cDEGs of ZEN exposure and NAFLD. (E) The biological process of GO enrichment analysis of cDEGs of ZEN exposure and NAFLD. (F) The KEGG enrichment analysis of cDEGs of ZEN exposure and NAFLD. (G) PPI analysis of cDEGs of ZEN exposure and NAFLD. Data are expressed as mean ± SD. Statistical significance was determined by two-sided unpaired Student’s *t*-test.

Based on previous experimental results and literature, we believe that there is a potential connection between ZEN exposure and the occurrence of NAFLD. In order to further explore the potential connection between ZEN exposure and NAFLD, we conducted bioinformatics analysis of the two using the latest database. We utilized the GEO platform to screen a relevant database GSE89632 which containing NAFLD clinical samples (Fig. 5A). After normalizing, filtering, and crossing the data, 346 differentially expressed genes (DEGs) were identified (Fig. 5B,C). The DEGs of ZEN-exposure were collected from GeneCards, the Comparative Toxicgenomics Database and our results, totally 1103 DEGs were identified. Finally, totally 36 cross-differentially expressed genes (cDEGs) associated with both ZEN exposure and NAFLD were identified (Fig. 5D).

**Fig. 6:**
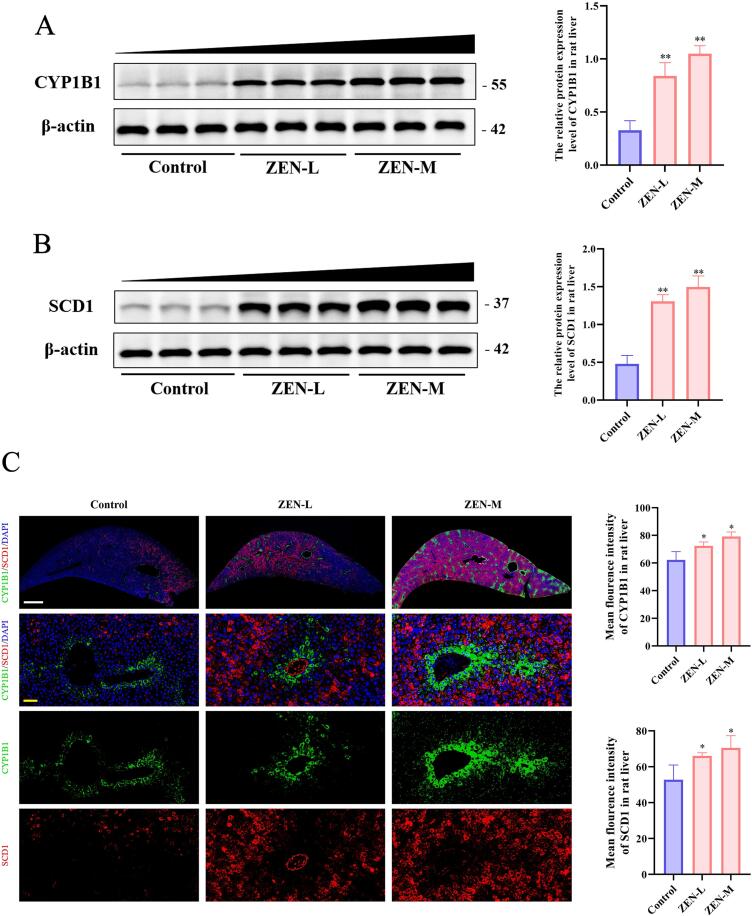
Western blotting and IF of CYP1B1 and SCD1 in control, ZEN-L and ZEN-M group. (A) Relative protein expression level of CYP1B1 in control, ZEN-L and ZEN-M group. (B) Relative protein expression level of SCD1 in control, ZEN-L and ZEN-M group. (C) Representative images of IF staining of CYP1B1 and SCD1 in control, ZEN-L and ZEN-M group. White scale: 1000 μm; yellow scale:50 μm. Data are expressed as mean ± SD. Statistical significance was determined by two-sided unpaired Student’s *t*-test. n = 3–6. *p < 0.05, **p < 0.01 as compared to the control group.

We conducted GO, KEGG, and PPI analyses on 36 cDEGs. The biological process of GO enrichment analysis of the cDEGs (Fig. 5E) showed that ZEN exposure might increase the risk of NAFLD through cellular response to organic substance, lipid metabolic process and response to oxygen-containing compound, etc. KEGG pathway enrichment analysis of cDEGs (Fig. 5F) showed that ZEN exposure might increase the risk of NAFLD through pathways in cancer, PI3K − Akt signaling pathway and Non − alcoholic fatty liver disease, etc. The PPI analysis of the cDEGs (Fig. 5G) showed that the top 5 genes with the highest scores included SCD1, CCL2, IL-1β, PTGS2 and MYC. Finally, western blot and IF analysis revealed that the ZEN exposure dose-dependently exhibited significantly elevated protein levels of CYP1B1 and SCD1 in rat livers (Fig. 6) (p < 0.01), further corroborating the findings from the omics analysis.

## 4 Discussion

As a global pollutant that widely pollutes food and feed, ZEN can cause liver toxicity to humans and animals. ZEN induces significant oxidative stress by down-regulating mRNA and protein levels of Nrf2-ARE in mouse liver (Long et al., 2016). Recent studies had shown that ZEN exposure leads to hepatic inflammatory responses and oxidative stress via activation of NF-κB phosphorylation and inhibition of SIRT1-Nrf2 (Sun et al., 2023). In mouse liver, ZEN causes liver injury and apoptosis through activation of endoplasmic reticulum stress and MAPK signalling pathway (Wu et al., 2023). Previous studies had shown that ZEN significantly activated endoplasmic reticulum stress and upregulated the expression of Phase I/II Enzymes in HepG2 cell (Yoon et al., 2019). Our study showed that ZEN not only caused liver toxicity in rats, but also significantly affected the expression of endoplasmic reticulum related proteins, and the key DEPs identified after analysis (CYP1B1, SCD1, SOAT1 and SOAT2) were important biological enzymes located in the endoplasmic reticulum. Therefore, we believe that the significant increase in endoplasmic reticulum related enzymes caused by ZEN exposure may be a key factor in ZEN's increased susceptibility to NAFLD.

Benzo[a]pyrene led to Metabolic dysfunction-associated steatotic liver disease by promoting hepatic lipid accumulation through the CYP1B1-mediated suppression of lipophagy (Bu et al., 2024). Existing literature had demonstrated that ZEN, as an estrogen-like exogenous pollutant, significantly upregulates the level of the key estrogen-metabolizing enzyme CYP1B1 in MCF-7 cells (Malekinejad et al., 2023, Yu et al., 2004). Our study found that CYP1B1-SCD1 is a key target for ZEN-induced lipid accumulation in rat liver, we hypothesize that ZEN exposure exerts its exogenous estrogenic effects in rat liver, leading to a significant upregulation of the key estrogen-metabolizing enzyme CYP1B1. This subsequently activates the CYP1B1-SCD1 axis, promoting abnormal lipid accumulation in rat liver. Abnormal lipid accumulation may lead to a series of pathological changes such as insulin resistance and initiate or accelerate the onset and progression of NAFLD.

Mycotoxins have been widely recognized as a risk factor for NAFLD (Cheung et al., 2020, Kawahara et al., 2022 Oct).The activation of CYP1B1-SCD1 plays a crucial role in the occurrence and development of NAFLD (Bock, 2020, Lee et al., 2021). Our experiment explored the changes in rat liver proteins caused by ZEN exposure through proteomics, and explored the potential relationship between ZEN exposure and the occurrence and development of NAFLD through bioinformatics analysis based on DEPs and the latest database; however, the results obtained through this correlation-based analytical approach still require further experimental validation. We preliminarily speculate that ZEN may increase the susceptibility of NAFLD by activating CYP1B1-SCD1, providing a theoretical basis for ZEN's health risk assessment and prevention and treatment of NAFLD.

However, this study has only identified the effect of ZEN exposure on hepatic lipid accumulation in rats, lacking mechanistic validation experiments. Future research will investigate the temporal progression of key events leading to liver pathology (Ghallab et al., 2021), while utilizing CYP1B1 knockout models (both in vivo and in vitro) and CYP1B1 pharmacological inhibition models to clarify the key targets linking ZEN exposure to NAFLD (Tung et al., 2024, Bushkofsky et al., 2016). Additionally, more epidemiological data are needed to explore the association between dietary ZEN exposure and the development of NAFLD in humans.

## 5 Conclusion

ZEN exposure can lead to abnormal liver lipid metabolism, and long-term lipid abnormalities may induce and promote NAFLD. Current research has determined that environmental factors, including mycotoxin-exposure, may induce NAFLD, but the relationship between ZEN exposure and NAFLD has not yet clear. In this study, we established an animal model of ZEN-induced hepatic lipid accumulation, and identified key targets of ZEN exposure induced lipid accumulation through proteomics. In addition, we speculated on the potential link between ZEN exposure and NAFLD through bioinformatics analysis based on DEPs and the latest database results. The research results indicated that ZEN exposure significantly increased the expression levels of the SOAT family, CYP1B1, and SCD1 proteins in the rat liver. Based on bioinformatics analysis and animal experiments, we propose that ZEN exposure may increase susceptibility to NAFLD by activating CYP1B1-SCD1.

## CRediT authorship contribution statement

**Haonan Ruan:** Writing – original draft, Methodology, Data curation, Investigation, Software, Validation. **Jing Zhang:** Data curation, Visualization, Formal analysis. **Yunyun Wang:** Writing – review & editing, Data curation, Software. **Dan Zhang:** Supervision, Writing – review & editing. **Jiaoyang Luo:** Resources, Supervision, Conceptualization, Writing – review & editing. **Meihua Yang:** Project administration, Supervision, Funding acquisition, Writing – review & editing.

## Declaration of competing interest

The authors declare that they have no known competing financial interests or personal relationships that could have appeared to influence the work reported in this paper.
