# Supplementary material for: Zearalenone exposure may increase the risk of non-alcoholic fatty liver disease by activating CYP1B1-SCD1
Source: Curr Res Toxicol. 2025 Dec 13;10:100277. doi: 10.1016/j.crtox.2025.100277 (PMC12771349; doi:10.1016/j.crtox.2025.100277)
Supplement: Supplementary Data 1 [file mmc1.docx]

**Supplementary Table Title**

Table S1 DEPs of ZEN vs. control. in Rat liver

**Table S1**

| **Proteins** | **Control-1** | **Control-2** | **Control-3** | **ZEN-1** | **ZEN-2** | **ZEN-3** | **Log_2_FC*** | **p-value*** |
| --- | --- | --- | --- | --- | --- | --- | --- | --- |
| Q3MIE7 | 6.6475 | 8.3718 | 14.1864 | 0.1697 | 0.2103 | 0.1841 | -5.694 | 0.0018 |
| RPB7 | 0.7741 | 0.9118 | 1.4529 | 0.1939 | 0.1984 | 0.2825 | -2.2178 | 0.0043 |
| A0A0G2KAJ7 | 0.2708 | 0.2821 | 0.1532 | 0.0896 | 0.0492 | 0.0504 | -1.9001 | 0.0089 |
| PCKGC | 19.2142 | 26.9306 | 29.23 | 4.9018 | 9.0193 | 6.5913 | -1.8776 | 0.005 |
| D3ZLC1 | 0.4939 | 0.6932 | 0.769 | 0.1955 | 0.2132 | 0.165 | -1.7696 | 0.0035 |
| CP2DQ | 38.0474 | 24.5678 | 54.2312 | 15.1856 | 9.3706 | 15.5976 | -1.541 | 0.0248 |
| G3V7Q7 | 1.3518 | 2.2987 | 2.92 | 0.4438 | 1.0555 | 0.8456 | -1.4865 | 0.04 |
| A0A0G2K2P4 | 0.6546 | 0.4856 | 0.5239 | 0.233 | 0.1903 | 0.1878 | -1.4452 | 0.0012 |
| RMD3 | 2.8526 | 2.8593 | 1.9369 | 1.0085 | 0.7303 | 1.2478 | -1.3567 | 0.0102 |
| ALD1 | 96.5308 | 173.115 | 172.1046 | 75.2737 | 48.7458 | 63.9239 | -1.2329 | 0.029 |
| CLC4F | 0.7009 | 0.9148 | 1.1176 | 0.3815 | 0.417 | 0.4191 | -1.1667 | 0.0234 |
| HUTH | 34.0863 | 43.1399 | 67.7138 | 29.8375 | 20.424 | 15.4961 | -1.1402 | 0.0472 |
| MEST | 0.12 | 0.0959 | 0.1528 | 0.052 | 0.072 | 0.045 | -1.1246 | 0.0156 |
| A0A0H2UHL2 | 5.9281 | 8.3178 | 4.8913 | 2.8578 | 2.2493 | 4.0703 | -1.0602 | 0.0336 |
| HEAT6 | 0.2262 | 0.2409 | 0.3326 | 0.1542 | 0.1356 | 0.1001 | -1.0363 | 0.0149 |
| AMPE | 3.7487 | 4.9498 | 7.0103 | 3.2297 | 2.4019 | 2.0914 | -1.0243 | 0.0407 |
| S22A7 | 0.3148 | 0.3567 | 0.3158 | 0.1562 | 0.2133 | 0.1204 | -1.0107 | 0.0413 |
| PSB7 | 0.8703 | 1.4549 | 1.5368 | 2.1876 | 2.9438 | 2.6119 | 1.0036 | 0.0401 |
| TOM22 | 0.1905 | 0.2158 | 0.2879 | 0.4919 | 0.4628 | 0.4413 | 1.008 | 0.0225 |
| RT07 | 0.0746 | 0.0999 | 0.0767 | 0.1449 | 0.1979 | 0.1647 | 1.014 | 0.0056 |
| BAF | 1.2296 | 1.2232 | 0.9279 | 1.8563 | 2.463 | 2.5602 | 1.025 | 0.0069 |
| Q4QQS7 | 0.818 | 1.084 | 1.3306 | 2.0161 | 2.5355 | 2.0357 | 1.027 | 0.0193 |
| D4A1Y5 | 0.2443 | 0.1601 | 0.2194 | 0.3575 | 0.5448 | 0.3705 | 1.0288 | 0.0187 |
| B1WC61 | 3.8858 | 3.9786 | 2.7673 | 7.5617 | 4.8718 | 9.3145 | 1.0325 | 0.0467 |
| G3V6F4 | 0.2762 | 0.3866 | 0.2645 | 0.6076 | 0.6482 | 0.6558 | 1.0438 | 0.022 |
| D3ZUT9 | 0.0345 | 0.0427 | 0.0329 | 0.0574 | 0.0791 | 0.0918 | 1.0514 | 0.0179 |
| A0A0G2JSL0 | 1.5638 | 1.7762 | 2.0421 | 3.4271 | 3.6715 | 4.102 | 1.0573 | 0.0023 |
| STX17 | 0.1606 | 0.1207 | 0.1435 | 0.2852 | 0.3029 | 0.2965 | 1.0583 | 0.0098 |
| MECR | 1.3347 | 1.3622 | 1.1838 | 2.0754 | 2.6989 | 3.3303 | 1.0624 | 0.0254 |
| TMLH | 0.1925 | 0.1452 | 0.1858 | 0.4224 | 0.331 | 0.3467 | 1.0713 | 0.0033 |
| M0R4K6 | 0.2153 | 0.1809 | 0.1144 | 0.4037 | 0.3276 | 0.3529 | 1.0863 | 0.0425 |
| ST1B1 | 17.328 | 15.2429 | 18.1269 | 33.2814 | 35.0728 | 39.4222 | 1.088 | 0.0005 |
| CR032 | 0.1284 | 0.1157 | 0.1598 | 0.2874 | 0.2225 | 0.3516 | 1.0929 | 0.0126 |
| GSTM1 | 81.0417 | 94.1028 | 123.2449 | 225.2912 | 160.9451 | 252.4444 | 1.0979 | 0.0145 |
| ECI1 | 19.1934 | 31.822 | 25.2028 | 41.7326 | 61.2158 | 60.642 | 1.1019 | 0.017 |
| D3ZV82 | 0.2662 | 0.2516 | 0.178 | 0.5273 | 0.4191 | 0.5503 | 1.105 | 0.0099 |
| ACSL4 | 2.8814 | 2.3007 | 2.657 | 5.4773 | 5.6751 | 5.7403 | 1.1076 | 0.0054 |
| D3ZI16 | 0.3016 | 0.3643 | 0.3788 | 0.6733 | 0.6554 | 0.927 | 1.1104 | 0.0074 |
| UD11 | 18.2882 | 18.7462 | 20.9828 | 42.0001 | 30.613 | 52.8118 | 1.1123 | 0.0345 |
| G3V7I6 | 0.8912 | 1.1089 | 1.2532 | 1.6349 | 2.2209 | 3.2128 | 1.1196 | 0.0427 |
| Q6T5E9 | 7.7754 | 9.5577 | 9.2199 | 16.3388 | 20.2759 | 21.2839 | 1.1247 | 0.0021 |
| HSBP1 | 0.178 | 0.1318 | 0.1076 | 0.3486 | 0.2962 | 0.2656 | 1.1255 | 0.0164 |
| D3ZJ32 | 0.0687 | 0.1191 | 0.1182 | 0.2402 | 0.2269 | 0.2061 | 1.1375 | 0.0397 |
| B1WBY1 | 0.2528 | 0.3437 | 0.2985 | 0.5834 | 0.636 | 0.7718 | 1.1537 | 0.0028 |
| B2GV41 | 0.212 | 0.2675 | 0.3604 | 0.6493 | 0.6185 | 0.6311 | 1.1769 | 0.031 |
| F1M265 | 0.1124 | 0.1094 | 0.1745 | 0.3678 | 0.2007 | 0.3511 | 1.2146 | 0.0307 |
| A0A0G2K6B2 | 0.2007 | 0.1498 | 0.3196 | 0.6182 | 0.4278 | 0.5104 | 1.2159 | 0.0393 |
| HYEP | 33.9166 | 37.1785 | 47.1145 | 107.1812 | 69.622 | 98.3397 | 1.2188 | 0.0086 |
| D4ACK1 | 0.1962 | 0.1173 | 0.1754 | 0.371 | 0.4038 | 0.3678 | 1.2247 | 0.0272 |
| ARK73 | 4.0182 | 5.0314 | 7.4644 | 13.1793 | 11.2962 | 14.5597 | 1.2411 | 0.026 |
| G3V8L9 | 0.1341 | 0.0998 | 0.0884 | 0.2005 | 0.2039 | 0.3645 | 1.2545 | 0.0295 |
| DHC24 | 0.4587 | 0.6096 | 0.3606 | 0.8017 | 1.1925 | 1.416 | 1.2549 | 0.0192 |
| Q7TP42 | 0.381 | 0.4923 | 0.5595 | 1.3914 | 1.0152 | 1.045 | 1.2685 | 0.0045 |
| INO1 | 0.1565 | 0.1943 | 0.3022 | 0.4788 | 0.5797 | 0.5235 | 1.2766 | 0.0334 |
| PGPI | 0.3409 | 0.3707 | 0.3811 | 0.8238 | 0.9808 | 0.8568 | 1.2843 | 0.0004 |
| A0A0G2K828 | 0.1159 | 0.14 | 0.0876 | 0.2268 | 0.2632 | 0.3599 | 1.3069 | 0.0093 |
| CRYM | 1.1307 | 1.3111 | 2.0447 | 2.7977 | 2.9216 | 5.5314 | 1.3263 | 0.0356 |
| A0A0G2JXI4 | 0.1778 | 0.1548 | 0.3089 | 0.6089 | 0.5192 | 0.4919 | 1.3365 | 0.0352 |
| TBG1 | 0.0511 | 0.0565 | 0.0445 | 0.109 | 0.1273 | 0.1568 | 1.3698 | 0.003 |
| Q6IRJ7 | 0.5223 | 0.3175 | 0.3229 | 0.8496 | 1.2438 | 0.9177 | 1.3729 | 0.011 |
| D4ABN3 | 0.0483 | 0.0648 | 0.0949 | 0.1749 | 0.2585 | 0.1092 | 1.3834 | 0.0446 |
| Q6IMX8 | 9.2018 | 11.8566 | 10.4847 | 20.4877 | 32.3423 | 31.8938 | 1.4254 | 0.011 |
| AS3MT | 3.3813 | 6.0563 | 2.6119 | 8.0841 | 9.0209 | 16.156 | 1.4648 | 0.036 |
| A0A0G2K0B0 | 0.1305 | 0.2465 | 0.2647 | 0.6463 | 0.4144 | 0.7177 | 1.4707 | 0.0238 |
| D3ZPF0 | 0.0534 | 0.0642 | 0.1182 | 0.1857 | 0.326 | 0.1742 | 1.541 | 0.0267 |
| Q5U2U8 | 0.1156 | 0.1619 | 0.1729 | 0.5434 | 0.2547 | 0.5738 | 1.6067 | 0.0377 |
| B2GV01 | 0.3506 | 0.4635 | 0.4494 | 1.0274 | 1.3467 | 1.4776 | 1.6082 | 0.0016 |
| O35849 | 0.0585 | 0.0515 | 0.1181 | 0.2152 | 0.2449 | 0.2495 | 1.6375 | 0.0393 |
| Q4KM38 | 0.3237 | 0.43 | 0.6123 | 1.1616 | 1.912 | 1.2231 | 1.6532 | 0.0095 |
| CP1B1 | 0.0922 | 0.1115 | 0.0403 | 0.3314 | 0.3103 | 0.2519 | 1.873 | 0.0402 |
| UDB15 | 1.425 | 1.8225 | 1.2732 | 4.0426 | 3.9686 | 9.4462 | 1.9492 | 0.0344 |
| G3V6Y6 | 0.0719 | 0.0321 | 0.0814 | 0.3341 | 0.3387 | 0.1417 | 2.1359 | 0.0225 |
| F1LR92 | 0.3027 | 0.2348 | 0.5123 | 1.5883 | 1.4976 | 1.6694 | 2.1793 | 0.0193 |
| AL3B1 | 0.1102 | 0.1028 | 0.1094 | 0.5228 | 0.4741 | 0.4684 | 2.1841 | 0 |
| TINAL | 0.1991 | 0.2707 | 0.1562 | 0.9064 | 1.0471 | 1.0254 | 2.2506 | 0.0065 |
| Q5HZE3 | 0.0526 | 0.0813 | 0.0718 | 0.2189 | 0.498 | 0.4121 | 2.4567 | 0.0095 |
| G3V6J2 | 0.0728 | 0.0573 | 0.0434 | 0.5132 | 0.5085 | 0.6227 | 3.1386 | 0.0158 |
| SRC8 | 0.3212 | 0.2001 | 0.3942 | 2.8527 | 1.1451 | 4.0646 | 3.2453 | 0.0013 |
| AKC1H | 0.0099 | 0.0071 | 0.0081 | 0.1263 | 0.0872 | 0.0777 | 3.5301 | 0.0004 |
| DHI1 | 1.8813 | 0.3975 | 0.8615 | 3.0018 | 16.4163 | 21.897 | 3.7177 | 0.0364 |
| SCD1 | 0.0589 | 0.0835 | 0.1366 | 1.0285 | 3.0161 | 0.53 | 4.0353 | 0.0208 |
| G3V8B0 | 0.017 | 0.0071 | 0.0114 | 38.5652 | 30.9784 | 34.8408 | 11.5182 | 0.0005 |

*|Log_2_FC| > 1.0, p-value < 0.05 were considered statistically significant.
